# Improvement of Nicotine Removal and Ethanol Fermentability From Tobacco Stalk by Integration of Dilute Sulfuric Acid Presoak and Instant Catapult Steam Explosion Pretreatment

**DOI:** 10.3389/fbioe.2021.763549

**Published:** 2021-10-29

**Authors:** Hongsen Zhang, Chenqing Fu, Tianbao Ren, Hui Xie, Guotao Mao, Zhimin Wang, Fengqin Wang, Andong Song

**Affiliations:** ^1^ Key Laboratory of Enzyme Engineering of Agricultural Microbiology, College of Life Science, Ministry of Agriculture, Henan Agricultural University, Zhengzhou, China; ^2^ College of Tobacco, Henan Agricultural University, Zhengzhou, China; ^3^ College of Science, Henan Agricultural University, Zhengzhou, China

**Keywords:** tobacco stalk, nicotine, dilute sulfuric acid presoak, instant catapult steam explosion (ICSE), enzymatic hydrolysis, ethanol fermentation

## Abstract

The nicotine from tobacco stalk showed obvious inhibitory effect on the activity of cellulase and fermentability of microorganisms, which seriously hinders the utilization of tobacco stalk. Dilute sulfuric acid presoak of tobacco stalk was used to enhance the performance of instant catapult steam explosion (ICSE) for tobacco stalk pretreatment. The presoak was beneficial to break the recalcitrant structure of tobacco stalk, reduce nicotine content to relieve the inhibition on the activity of cellulase and metabolism of microorganisms, and promote the performance of enzymatic hydrolysis and ethanol fermentation. The optimized 0.8% sulfuric acid (w/w) presoak–integrated ICSE pretreatment resulted in 85.54% nicotine removal from tobacco stalk; meanwhile, the total sugar concentration from enzymatic hydrolysis of pretreated tobacco stalk increased from 33.40 to 53.81 g/L (the ratio of dry tobacco stalk to water was 1:8, w/w), ethanol concentration increased 103.36% from 5.95 to 12.10 g/L in flask, compared with separate ICSE pretreatment. Finally, the ethanol concentration achieved the highest 23.53 g/L in a 5-L fermenter with the ethanol yield from the glucose of tobacco stalk hydrolysate achieving 71.40% by increasing the solid loading of the tobacco stalk in the enzymatic hydrolysis process (the ratio of dry tobacco stalk to water was 1:4, w/w). These results achieved the expected purpose of efficient utilization of discarded tobacco stalk.

## Introduction

With the vigorous development of the world economy, the environment pollution and nonrenewable resources consumption have become a severe challenge to the human society; more and more attention has been focused on new renewable and environment-friendly energy; and relevant research has become a mainstream of scientific community (Herbert and Krishnan, 2016). As a clean and renewable energy, bioethanol showed many significant advantages such as being green and environment-friendly, having low manufacturing cost, having wide availability, and so on (Zhou et al., 2019). In the foreseeable future, it is expected to gradually replace the diminishing fossil fuels, such as oil, coal, and natural gas (Morales et al., 2015).

The planting area and annual output of tobacco in China ranked first in the world in recent years (Qin et al., 2018). Tobacco stalk as the main waste of the tobacco industry had resulted in a series of environmental problems (Wang et al., 2019). The resource utilization of tobacco stalk has become an urgent problem to be solved. As a kind of lignocellulosic biomass, tobacco stalk could be used to produce many valuable bio-based products, such as bioethanol by biorefinery. Tobacco stalk was mainly composed of cellulose, hemicellulose, and lignin, and their content in tobacco stalk was approximately 45, 15, and 20%, respectively (Su et al., 2016). In addition, tobacco stalk also contains nicotine, solanesol, pectin, protein, amino acid, organic acids, sugar, and other compounds (Akpinar et al., 2010; Huang et al., 2019). Nicotine is the main alkaloid of tobacco, which accounts for over 95% of the total alkaloid content of tobacco (Henry et al., 2019). Meanwhile, nicotine is both an addictive and a toxic substance, which can inhibit the activity of biological enzymes and the growth of microorganisms, and finally can go against tobacco stalk efficient utilization (Swanson et al., 1994; Zhong et al., 2010; Falco and Bevins, 2015). Therefore, removal of nicotine is as important as the breaking of the compact structure for the bioconversion of tobacco stalk. Pretreatment of tobacco stalk was the precondition for the conversion of cellulose and hemicellulose of tobacco stalk into fermentable sugar. The pretreatment methods of lignocellulose at present mainly includes physical, chemical, biological methods, or a mixture of these methods (Cai et al., 2016; Sun et al., 2020; Zhang et al., 2020). Instant catapult steam explosion (ICSE) has become an efficient, economic, and environmental pretreatment technology, depending on its short pretreatment time, low energy consumption, and zero pollution to the environment (Wang et al., 2020; Xie et al., 2020).

The mechanism of ICSE is to break the compact physical structure by the energy from high pressure release to barometric pressure and high temperature cool down, not more than 100°C, instantly (Yu et al., 2012). The cellulose, hemicellulose, and lignin were all hydrolyzed partly and separated in this ICSE process, which benefits the subsequent cellulosic ethanol fermentation. However, a separate ICSE pretreatment could not reduce nicotine to a satisfactory concentration, and this pretreatment method had to be improved. As an alkaloid, nicotine easily combines with acid to form salt, which is more easily dissolved in water and excluded from tobacco stalk (Henry et al., 2019). In addition, acid can change the surface chemical and cellulose crystalline structure of the tobacco stalk (Liang et al., 2021). The integrated dilute acid presoak and ICSE technology was used for tobacco stalk pretreatment in this study, where the compact crystal structure of the tobacco stalk was broken and more than 80% of the nicotine removed, which could improve the enzymatic hydrolysis of the tobacco stalk and fermentability of bioethanol. This study provides a kind of lignocellulosic ethanol production process from tobacco stalk with a potential for industrial applications, and also a theoretical basis for the diversified utilization of tobacco stalk to produce more bio-based products.

## Materials and Methods

### Feedstocks

Tobacco stalk was harvested from a tobacco test field at the Henan Agricultural University (Xuchang campus) in the fall of 2018. After collection, the biomass was milled coarsely using a hammer crusher and screened through a mesh with the circle diameter of 10 mm. The milled tobacco stalk was washed to remove field dirt, stones, and metals, and then dried to constant weight at 105°C in an air oven. The composition of the tobacco stalk after pre-handling treatment contained 41.64% cellulose, 14.96% hemicellulose, 15.74% lignin, and 7.43% ash determined by the method described in the NREL protocols (Sluiter et al., 2008). The nicotine content of the tobacco stalk was 0.83% (w/w), which was measured by HPLC (Dash and Wong, 1996).

### Enzyme

Commercial cellulase was purchased from Hunan Youtell Biochemical Co., Yueyang, Hunan, China. The filter paper activity was 214 FPU/g cellulase, according to the National Renewable Energy Laboratory (NREL) protocol LAP-006 (Adney and Baker, 1996).

### Strains and Mediums

*Issatchenkia orientalis* HN-1 was isolated from decayed tobacco stalk at our lab and stored at Key Laboratory of Enzyme Engineering of Agricultural Microbiology.

*Saccharomyces cerevisiae* 1308 was obtained from Henan Tianguan Group Co., Ltd., Nanyang city, Henan province, China.

Strain activation medium: 30 g of glucose, 10 g of yeast extract, 20 g of peptone, 20 g of agar in 1 L deionized water.

Seed medium: 30 g of glucose, 10 g of yeast extract, 20 g of peptone in 1 L deionized water.

Fermentation medium for ethanol production (synthetic medium): 50 g of glucose (or tobacco stalk hydrolysate), 3 g of yeast extract, 5 g of peptone, 0.2 g of urea, 0.1 g of (NH_4_)_2_HPO_4_ in 1 L deionized water, pH 5.5.

Fermentation medium for ethanol production (tobacco stalk hydrolysate): 3 g of yeast extract, 5 g of peptone, 0.2 g of urea, 0.1 g of (NH_4_)_2_HPO_4_ in 1 L tobacco stalk hydrolysate, pH 5.5.

All the medium of *I. orientalis* HN-1 and *S. cerevisiae* 1308 were the same in this study.

### Activation and Large Scale of Strain

The culture solution of *I. orientalis* HN-1 (or *S. cerevisiae* 1308) was maintained at −80°C in a freezer in 2-ml stock vials containing 30% (v/v) glycerol solution. 100 μL of liquid from one stock vial was inoculated onto a plate with activation medium by spread plate method and cultured at 38°C for 48 h. A whole colony of *I. orientalis* HN-1 growing on slant medium was inoculated into a 250-ml flask containing 50 ml seed medium for seed culture at 38°C, 180 rpm for 12 h. A whole colony of *S. cerevisiae* 1308 growing on a slant medium was inoculated into a 250-ml flask containing 50 ml seed medium for seed culture at 30°C, 150 rpm for 16 h.

### The Effect of Nicotine on Activity of Cellulase

The carboxymethyl cellulase (CMC) activity of cellulase was measured by the DNS method (Eveleigh et al., 2009). The filter paper activity of cellulase was measured by the National Renewable Energy Laboratory (NREL) protocol LAP-006 (Adney and Baker, 1996). 0.1, 0.2, 0.3, and 0.5% (w/w) nicotine were added into the enzymatic reaction system.

### Dilute Sulfuric Acid Presoak

The tobacco stalk was presoaked into a series of dilute sulfuric acid solution concentrations (0.2–1.0%, w/w, on dry tobacco stalk weight) at a ratio of the solid (the dry materials) to the liquid (the sulfuric acid solution) of 1:10 (w/w) for 12 h at room temperature. The presoak slurry was filtered using double gauze; the solid residue was collected and dried, until the moisture was maintained at 60°C.

### Instant Catapult Steam Explosion Pretreatment

The ICSE method was used for pretreating the tobacco stalk feedstock in this study (Yu et al., 2012). 100 g fresh dry tobacco stalk (or presoaked dry tobacco stalk) was fed into a pretreatment reactor directly, and the pretreatment was operated at 2.0 MPa for 2.5 min (the optimized condition obtained from previous studies). The pretreated tobacco stalk after ICSE contained approximately 40% water of dry solid matter (w/w).

### Washing of the Pretreated Material

The pretreated material was washed by tenfold volume of water at 60°C for 1 h, and then the solid residue was collected by Buchner funnel filtration. After this operation was repeated, the solid residue was dried at 75°C in an oven until constant weight, then sealed in plastic bags and stored at room temperature until use.

### Enzymatic Hydrolysis

The pretreated tobacco stalk solution at a ratio of the solid (the dry materials) to the liquid (the deionized water) of 1:8 or 1:4 (w/w) was hydrolyzed at 50°C, 150 rpm, pH 4.8 for 48 h at the cellulase dosage of 15 FPU/g DM (dry materials). The hydrolysate slurry was centrifuged to remove the solids, then autoclaved, and filtered by filter paper before use.

### Ethanol Fermentation

The seed broth of *I. orientalis* HN-1 was inoculated into a 300-ml flask containing 240 ml fermentation medium with 10% (v/v) inoculation ratio at 38°C, stationary culture for 72 h. The seed broth of *S. cerevisiae* 1308 was inoculated into a 300-ml flask containing 240 ml fermentation medium with 10% (v/v) inoculation ratio at 30°C, stationary culture for 72 h. All flask cultures were carried out in triplicate. For the fermenter culture, the seed broth of *I. orientalis* HN-1 was inoculated into a 5- L fermenter containing 3 L fermentation medium with 10% (v/v) inoculation ratio at 38°C, pH 5.5, 100 rpm for 72 h.

### Analysis Methods

Samples were periodically taken, centrifuged, and filtrated through 0.22-μm filters before analysis. Glucose, xylose, and ethanol were measured by HPLC (LC-20 AD, refractive index detector RID-10A, Shimadzu, Kyoto, Japan) with Aminex HPX-87H column (Bio-Rad, Hercules, United States) at 65°C using the mobile phase of 5 mM H_2_SO_4_ at the flow rate of 0.6 ml/min. The sample size was 10 μL (Zhao et al., 2020). The concentration of nicotine was determined by HPLC (Thermo UltiMate 3000, UVVis detector, Thermo, Waltham, United States) with Inertsil ODS-3 C18 column (Shimadzu, Kyoto, Japan) at 35°C using the mobile phase of methanol–citrate phosphate buffer (15:85°v/v, pH 2.4 adjusted by addition of perchloric acid) at the flow rate of 0.7 ml/min. The column effluent was monitored at 260 nm with a UV-Vis spectrophotometric detector (Dash and Wong, 1996).

### Calculation of Ethanol Yield

Ethanol yield based on glucose of tobacco stalk hydrolysate was calculated by the following formula:

$$\mathrm{Ethanol}\;\mathrm{yield}\;\left(\%\right)=\frac{\left[C1\right]-\left[C0\right]}{0.511\times \left[Glucose\right]}\times 100\%,$$

where [*C*_1_] is the final ethanol concentration (g/L) of the fermentation broth at the end of fermentation; [*C*_0_] is the initial ethanol concentration (g/L) of the fermentation broth at the start of fermentation; [*Glucose*] is the initial glucose concentration (g/L) of the fermentation broth at the start of fermentation; 0.511 is the conversion factor for glucose to ethanol based on the stoichiometric biochemistry of yeast.

## Results and Discussion

### The Effect of Nicotine on Activity of Cellulase and Ethanol Productivity of Yeasts

Tobacco stalk as a bulk lignocellulosic biomass would have an important impact on biorefinery if it could be used for biochemical production efficiently. Previous research indicated that the tobacco stalk was more difficult to be used in biochemical production than common crop stalks (such as corn stover, wheat stalk, and so on) because of its high nicotine content. Nicotine from tobacco stalk has been shown to obviously inhibit the activity of glutathione reductase, aldehyde dehydrogenase, lactate dehydrogenase, and lipase (Gan and Zhuang, 2002; Erat et al., 2007; Trigo and Foll, 2016; Yalcin et al., 2018). However, the effect of nicotine on cellulase has been rarely reported.

**FIGURE 1:**
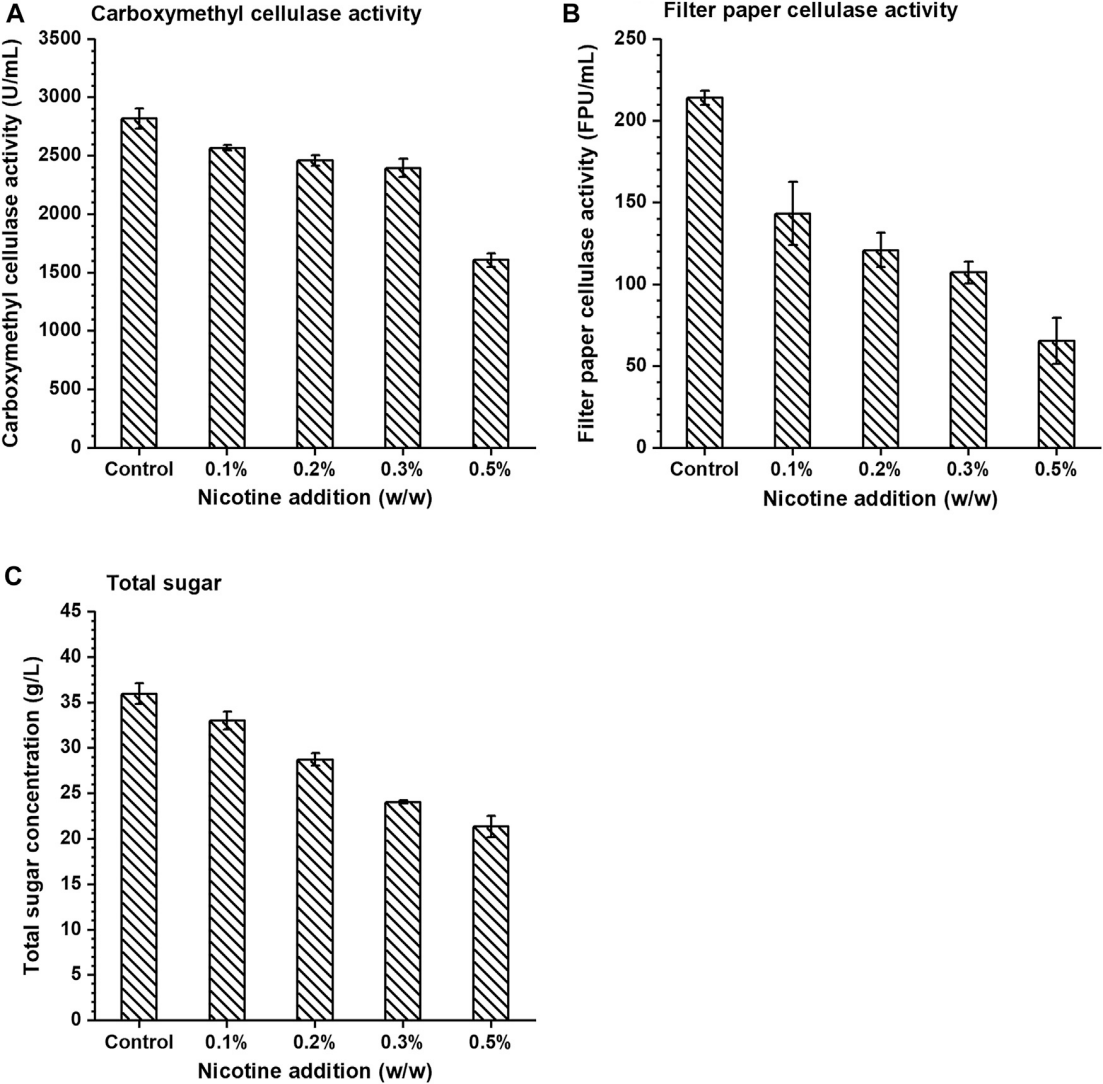
The effect of nicotine addition on the **(A)** carboxymethyl cellulase activity, **(B)** filter paper cellulase activity, and **(C)** total sugar concentration in enzymatic hydrolysis.

Three substrates (sodium carboxymethylcellulose, filter paper, and corn stover) were used to study the effects of nicotine on the activity of cellulase which was always used in biomass hydrolysis processes. In this study, cellulase activity was characterized by CMC activity and filter paper cellulase activity (FPA), and the results showed that the activity of cellulase decreased gradually with the increase of nicotine concentration (Figure 1). In the control group, CMC activity and FPA were 2820.99 U/ml and 214.12 FPU/ml, respectively, under zero nicotine addition. When nicotine addition reached 0.1% (w/v), CMC activity and FPA were 2567.90 U/ml and 143.29 FPU/ml, respectively, which decreased by 8.97 and 33.08% compared with the control group, respectively. The enzyme activity decreased more significantly with more nicotine addition, and when the addition was 0.5% (w/v), the CMC activity and FPA decreased by 42.99 and 69.44% compared with the control group, respectively. The results demonstrated that nicotine inhibited the activity of cellulase significantly.

Based on the above results, the effect of nicotine on enzymatic hydrolysis of pretreated corn stover (the ratio of dry corn stover to water was 1:10, w/w) by ICSE (2.0 MPa, 2.5 min) was subsequently verified. With the increase of nicotine concentration in the enzymatic hydrolysis system of corn stover, the concentration of total sugar (glucose, xylose, cellobiose, and arabinose, similarly hereinafter) showed a significant decrease compared with the control group (Figure 1C). The total sugar concentration in the hydrolysate reached the highest 35.99 g/L in the control group and gradually decreased in the range of 0.1–0.5% (w/v). When the nicotine concentration was 0.5% (w/v), the total sugar concentration reached the lowest 21.38 g/L which decreased by 40.59% compared with the control group. The trend demonstrated that nicotine had a significant inhibitory effect on the enzymatic hydrolysis of corn stover.

**FIGURE 2:**
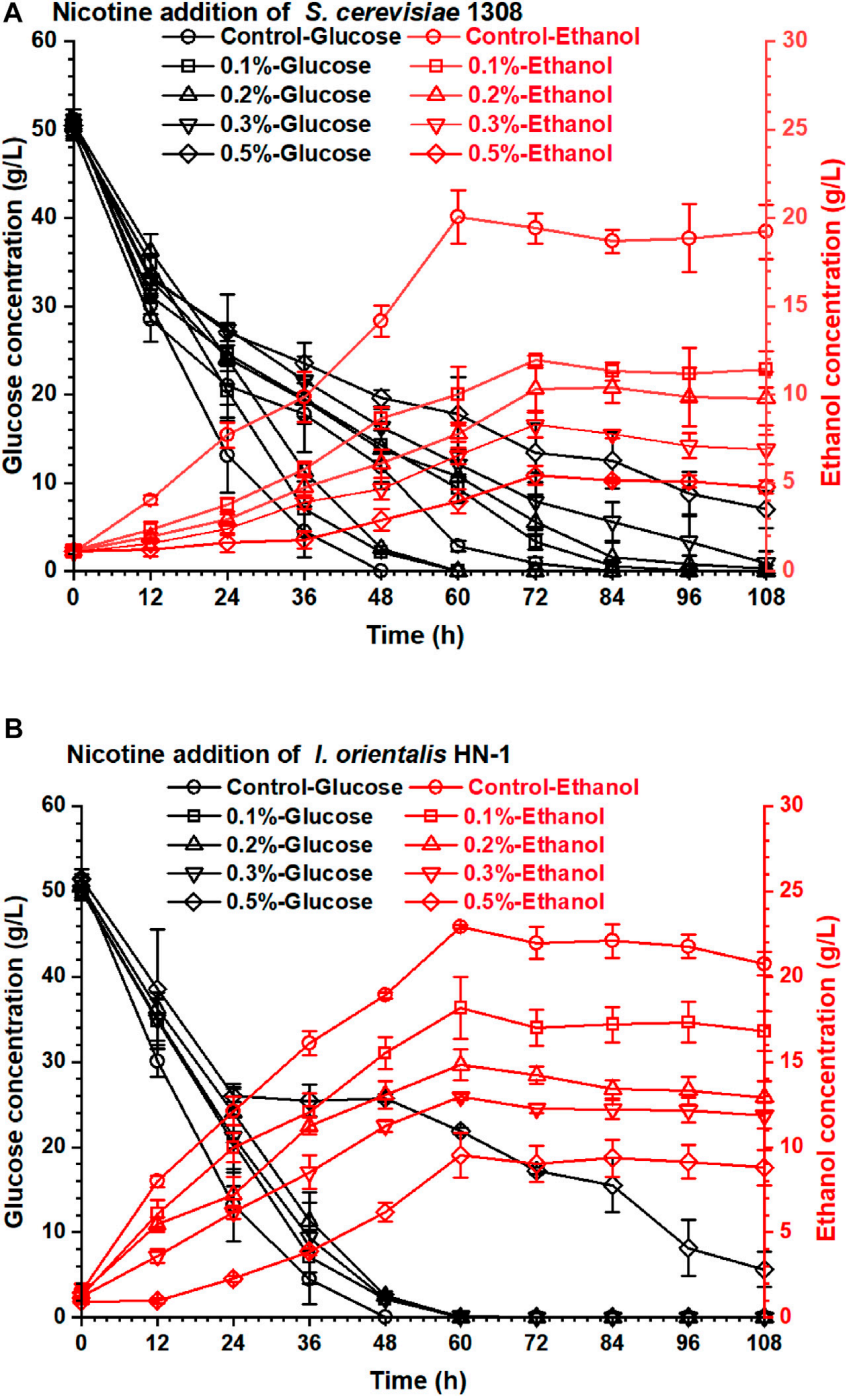
The effect of nicotine addition on the ethanol fermentability of yeasts: **(A)** *S. cerevisiae* 1308; **(B)** *I. orientalis* HN-1.

The nicotine not only inhibited the enzymatic activity of the cellulose system but also inhibited the fermentability of ethanol producer strains. *S. cerevisiae* 1308 is an excellent industrial ethanol producer which was obtained from the largest producer of fuel ethanol in China, but its fermentability decreased obviously under the nicotine stress (Figure 2A). Yeast (*I. orientalis* HN-1) isolated from decayed tobacco stalk showed excellent nicotine tolerance and was used in this research. The obtained highest ethanol concentration from *I. orientalis* HN-1 (22.94 g/L) in 60 h was similar to that from *S. cerevisiae* 1308 (20.05 g/L) under the control group (no nicotine addition in the fermentation medium). But when nicotine addition achieved just 0.1% (w/v), the ethanol concentration of *I. orientalis* HN-1 (18.17 g/L) decreased by 20.70%, and the ethanol concentration of *S. cerevisiae* 1308 (11.95 g/L) was even more inhibited, with 40.40% reduction compared with the control group. The results shown in Figure 2 indicate that nicotine had a serious impact on ethanol concentrations of both strains, especially on *S. cerevisiae* 1308. Therefore, the removal of nicotine from the tobacco stalk is essential in order to produce ethanol from discarded tobacco stalk. In addition, it is worth noting that *I. orientalis* HN-1 which was isolated from decayed tobacco stalk had a stronger nicotine tolerance than *S. cerevisiae* 1308 obviously, this laid a good foundation for subsequent high concentrations of ethanol production from tobacco stalk.

### The Effect of Separate Pretreatment Methods on Removal of Nicotine and Enzymatic Hydrolysis of Tobacco Stalk

**FIGURE 3:**
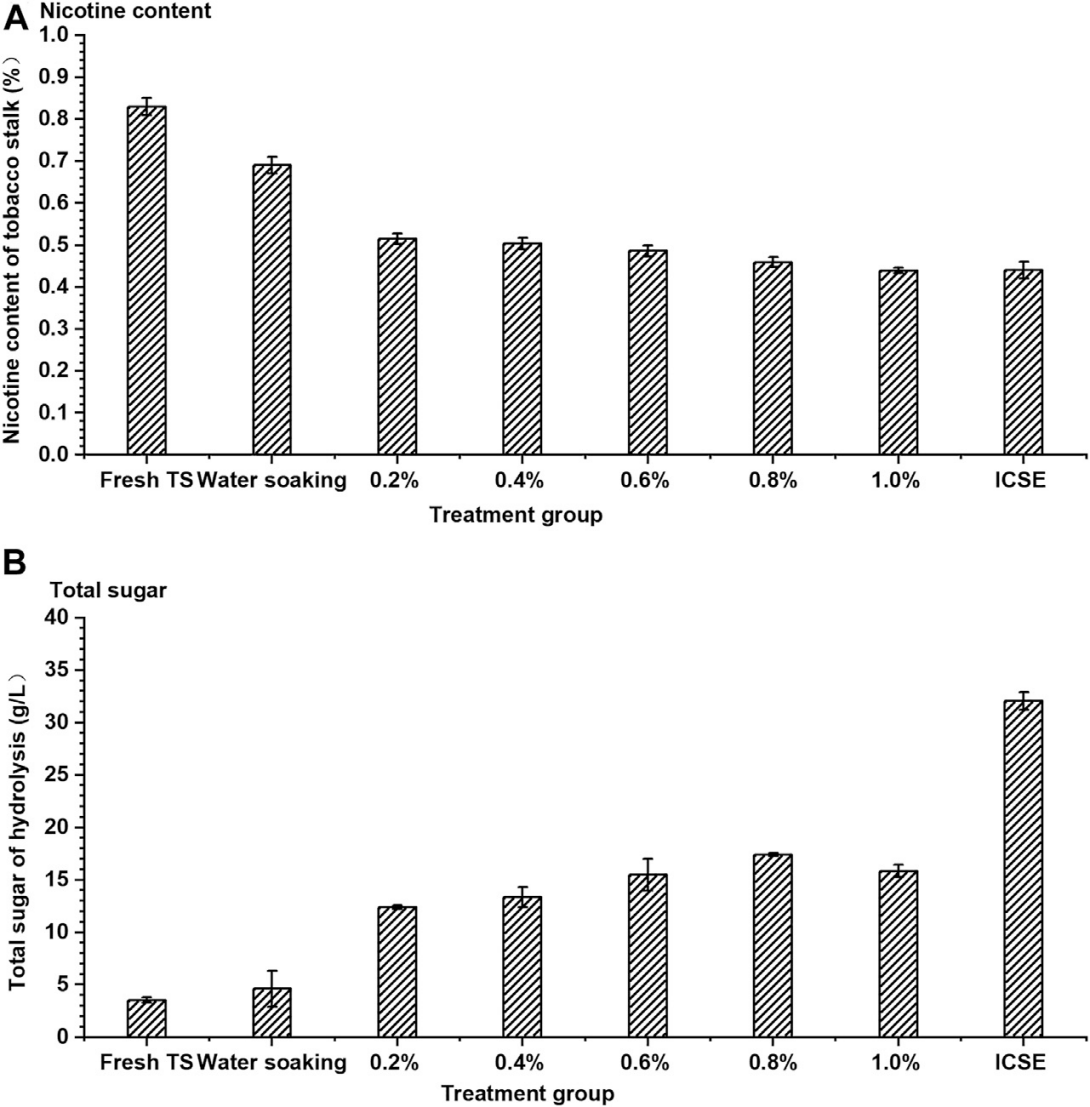
The **(A)** nicotine content of tobacco stalk and **(B)** total sugar after hydrolysis by different single pretreatment methods. TS means tobacco stalk; 0.2–1.0% means concentration of dilute sulfuric acid solution (0.2–1.0%, w/w, on dry tobacco stalk weight) at a ratio of the solid (the dry materials) to the liquid (the sulfuric acid solution) of 1:10 (w/w) for 12 h at room temperature. ICSE means instant catapult steam explosion pretreatment, 2.0 MPa, 2.5 min.

The existence of nicotine had seriously hindered the effective utilization of tobacco stalk, which might be the reason that tobacco stalks were rarely used to produce a bio-based chemical in previous studies. Therefore, efficient nicotine removal was particularly important for the development process of tobacco stalk utilization. A separate dilute sulfuric acid soaking pretreatment and a separate ICSE pretreatment were carried out in this experiment firstly. The nicotine removal rate and enzymatic saccharification efficiency of pretreated tobacco stalk were investigated and demonstrated in Figure 3. Dilute sulfuric acid solution could break the compact structure of tobacco stalk slightly; meanwhile, the nicotine from tobacco stalk as an alkaloid could combine with sulfuric acid easily. The nicotine content of pretreated tobacco stalk decreased gradually with increase in sulfuric acid adding dosage. The nicotine content achieved was the lowest at 0.44% (w/w) when the sulfuric acid adding dosage was 1.0% (w/v), and the nicotine removal rate was 46.99%. The nicotine always existed inside the tobacco stalk and was difficult to remove by water soaking. Yet, when 83.13% of the nicotine was retained in the tobacco stalk after water soaking, only 53.01% of nicotine was retained by water soaking after steam explosion pretreatment. This could be attributed to ICSE pretreatment that could break the internal structure of the tobacco stalk deeply, leading to more nicotine release into water that could be removed from pretreated tobacco stalk. The dilute sulfuric acid soaking could increase the nicotine removal rate significantly compared to water soaking; however, the nicotine removal rate was increased with the sulfuric acid concentration increasing inconspicuously. When the sulfuric acid concentration increased from 0.2 to 1.0% in the soaking process, the removal rates of nicotine were all similar to those of ICSE.

The removal rate of nicotine was an important indicator in this study, but the concentration of total sugar from enzymatic hydrolysis could not be ignored. Although dilute sulfuric acid soaking was effective with removal of nicotine, its contribution to the efficiency of enzymatic hydrolysis was limited compared with ICSE. The total sugar concentration from enzymatic hydrolysis (the ratio of dry pretreated tobacco stalk to water was 1:8, w/w) of the separate dilute sulfuric acid soaking pretreatment was 12.41–17.41 g/L and increased 252.56–394.60% compared with fresh tobacco stalk when sulfuric acid adding dosage was from 0.2 to 1.0% (w/v), but the highest total sugar concentration was approximately half that of ICSE. The total sugar concentration of ICSE was 32.05 g/L, but it still did not achieve satisfactory results because of a certain concentration of nicotine being present in the enzymatic hydrolysis system.

### Optimization of Integrated Pretreatment Condition for Tobacco Stalk

**FIGURE 4:**
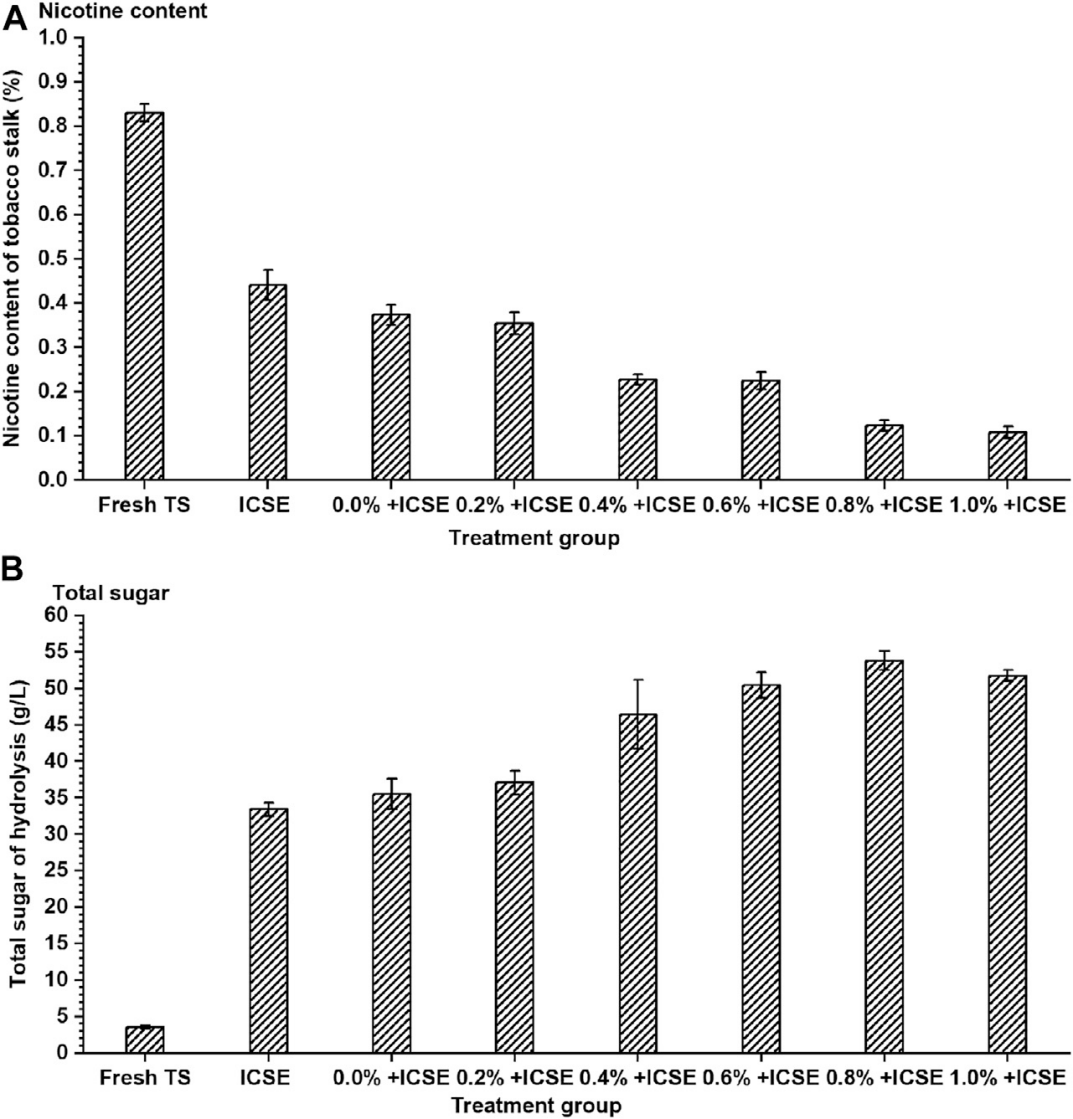
The **(A)** nicotine content of tobacco stalk and **(B)** total sugar after hydrolysis by different integrated pretreatment methods. TS means tobacco stalk; ICSE means instant catapult steam explosion pretreatment, 2.0 MPa, 2.5 min; 0.0%+ ICSE1.0%+ICSE means concentration of dilute sulfuric acid (0.0–1.0%, w/w, on dry tobacco stalk weight) in the presoak progress of integrated pretreatment.

Separate sulfuric acid pretreatment or steam explosion pretreatment for tobacco stalk showed unsatisfying enzymatic hydrolysis efficiency and nicotine removal rate, and the integrated pretreatment carried out achieved better performance (Figure 4). The nicotine content further significantly decreased from 0.37 to 0.11% (w/w) with sulfuric acid addition of presoak increasing from 0.0 to 1.0% in the integrated pretreatment (Figure 4A). Meanwhile, the total sugar concentration achieved the highest 53.81 g/L when sulfuric acid addition of presoak was 0.8% (w/w), which increased 74.58% compared with separate ICSE. When sulfuric acid addition of presoak increased continuously more than 0.8% (w/w), the total sugar concentration reduced a little and achieved 51.76 g/L at sulfuric acid dosage 1.0% of presoak (Figure 4B). High sulfuric acid addition of presoak might have enhanced the pretreatment strength too much to convert the sugar into inhibitors.

**FIGURE 5:**
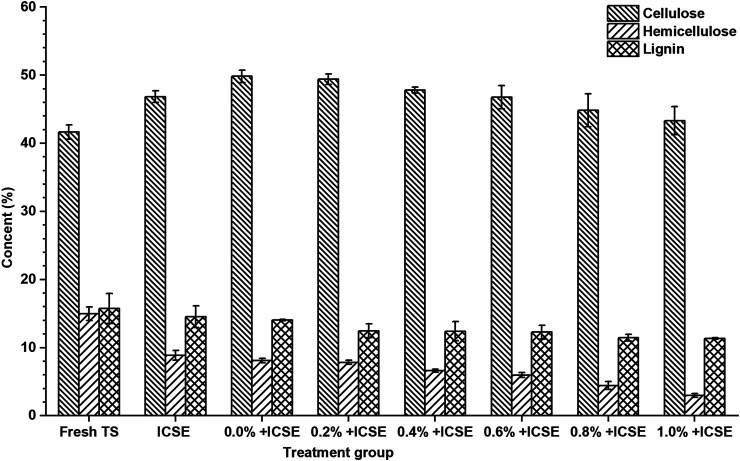
The cellulose, hemicellulose, and lignin content of pretreated tobacco stalk by different pretreatment methods.

Therefore, the compositions of pretreated tobacco stalk materials by integrated pretreatment and separate ICSE were measured (Figure 5). The results demonstrated that the hemicellulose and lignin content of pretreated tobacco stalk were both decreased which lead to the increase of cellulose content after both separate ICSE and integrated pretreatment compared with fresh tobacco stalk. But the cellulose and hemicellulose content decreased with the increase of sulfuric acid addition of presoak from 0.2 to 1.0% (w/v) in the integrated pretreatment, and this indicated that high-titer sulfuric acid enhances the hydrolysis of hemicellulose and cellulose, and the presence of free monosaccharides was more likely to produce inhibitors in the intense pretreatment process. Consider both the total sugar concentration and nicotine removal rate comprehensively, 0.8% (w/w) sulfuric acid dosage of presoak was used in subsequent ethanol fermentation.

### Ethanol Fermentation of Pretreated Tobacco Stalk

**FIGURE 6:**
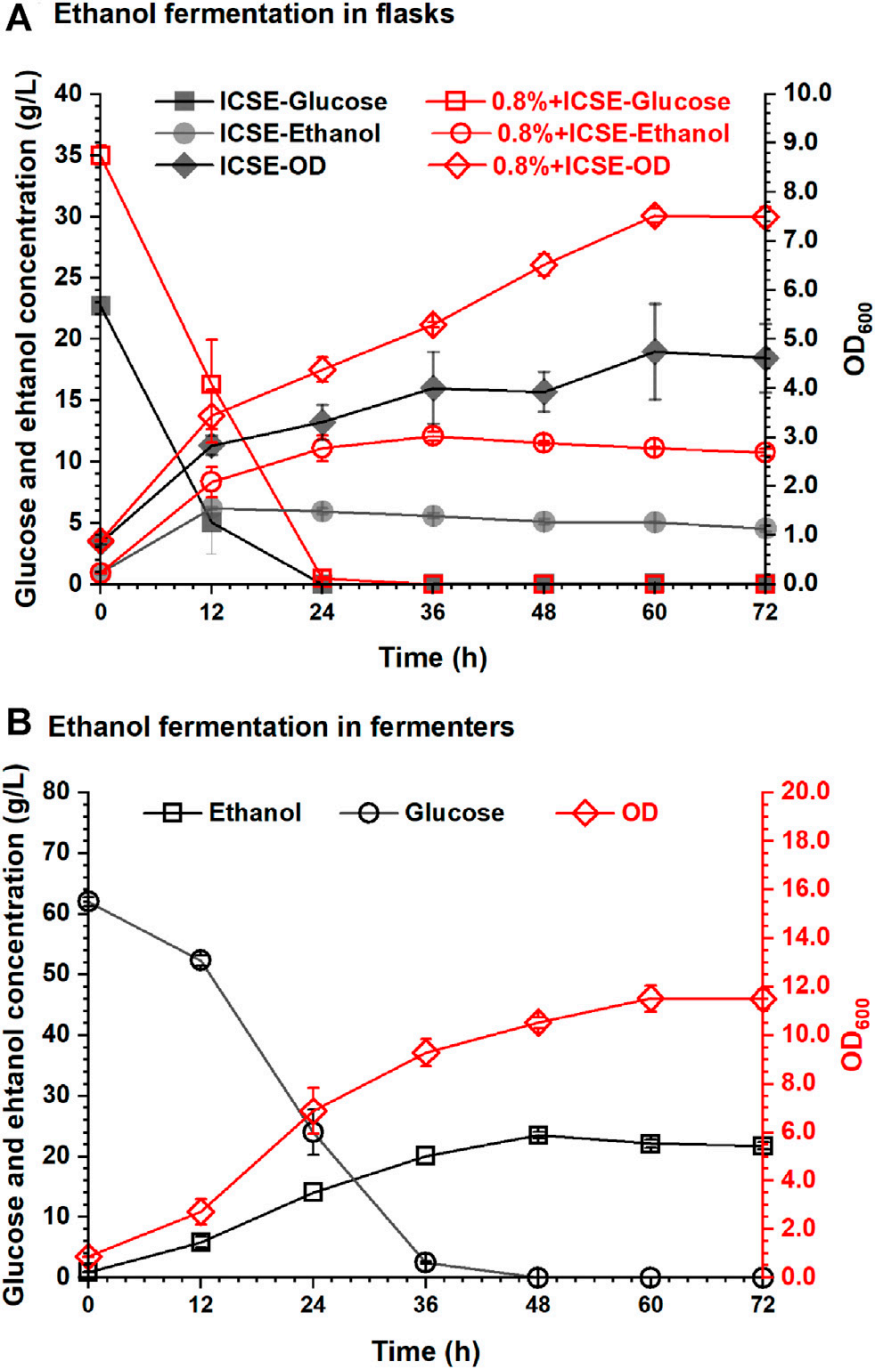
Ethanol fermentability of *I. orientalis* HN-1 by pretreated tobacco stalk: **(A)** in flasks; **(B)** in fermenters.

The ethanol fermentation by using tobacco stalk after integrated pretreatment with 0.8% (w/w) sulfuric acid presoak was carried out in this experiment, and the results are shown in Figure 6. The glucose concentration of tobacco stalk hydrolysate (the ratio of dry tobacco stalk to water was 1:8, w/w) through integrated pretreatment was 38.73 g/L, and the nicotine content was only 0.02% (w/v). Such a low concentration of nicotine would hardly affect the efficiency of enzymatic hydrolysis and *I. orientalis* HN-1. The ethanol concentration reached 12.10 g/L in 36 h, which increased 103.36% compared with 5.95 g/L of separate ICSE, the ethanol productivity was 0.31 g/L/h and the ethanol yield was 62.57%.

Based on the excellent nicotine removal performance of the integrated pretreatment, high solid loading of tobacco stalk could be implemented during enzymatic hydrolysis. A higher concentration of glucose could be obtained to produce higher concentration of ethanol. The glucose concentration of high solid loading tobacco stalk hydrolysate (the ratio of dry tobacco stalk to water was 1:4) through integrative pretreatment was 69.53 g/L, and the nicotine content was 0.05% (w/v). For better mixing and more precise control of pH, the next ethanol fermentation was carried out in the 5-L fermenter, and the results are shown in Figure 6B. With the increase in solid loading of tobacco stalk, the initial glucose concentration achieved was 62.00 g/L, and the growth of *I. orientalis* HN-1, consumption of glucose, and production of ethanol were affected to some extent in the first 12 h compared with the results shown in Figure 6A. But, the final ethanol concentration and yield for glucose of tobacco stalk hydrolysate were both increased and were 23.53 g/L and 71.40%, respectively, in 48 h.

### Discussion

In recent years, more attention has been paid to the effective utilization of tobacco residual. A combined hydrothermal (100°C, 20 min) and alkaline (2% CaO, w/v) pretreatment was used for bioethanol production from tobacco stalk, and the highest 5.43 g/L reducing sugar and 1.58 g/L ethanol were obtained in the fermentation process (Sophanodorn et al., 2020). Alkali pretreatment and acid pretreatment were used for ethanol production from tobacco stalk, and the highest 4.17 g/L ethanol was obtained under 2% NaOH pretreatment for 60 min (Guo et al., 2019). Yuan et al. compared ethanol fermentability of two different pretreatments (alkaline and acid-catalyzed steam pretreatments) for resource utilization of tobacco stalk, and the final ethanol concentration of alkaline pretreatment (14.2 g/L) was slightly higher than that of acid-catalyzed steam pretreatment (13.8 g/L) (Yuan et al., 2019). The integrated dilute sulfuric acid presoak and ICSE pretreatment technology were used in this study, which could enhance the degradation of hemicellulose in tobacco stalk and the removal of nicotine, and then weaken the inhibition of nicotine on the activity of cellulase in enzymatic saccharification process and fermentability of yeast strains. In this study, the highest ethanol concentration was 23.53 g/L, which was at a relatively high level compared with the current researches on the resource utilization of tobacco stalk.

In addition, it is worth noting that the important role of nicotine in ethanol fermentation by using tobacco stalk was studied in this work. Even though the low concentration of nicotine showed significant inhibition of cellulase and microorganisms, the ethanol concentration increased significantly after a large proportion of nicotine was removed in this integrated sulfuric acid presoak and ICSE pretreatment process. The reasons for insufficient ethanol yield (71.40%) might be because, although the nicotine in the tobacco stalk was almost removed out, there was still a variety of harmful substances (such as tar, benzopyrene, and so on) that could still inhibit the microorganisms. Although *I. orientalis* HN-1 showed strong tolerance to nicotine, the tolerance to other harmful substances remained to be studied. On the other hand, high-intensity pretreatment could produce a large number of common inhibitors (such as furfural, HMF, phenolic aldehyde, and so on), which could also inhibit the growth and ethanol fermentability of *I. orientalis* HN-1. In the future, some weak acids (such as organic acids) could be used for presoak to reduce the intensity in order to reduce the production of inhibitors. Dai et al. used furoic acid to assist hydrolysis of sugarcane bagasse, and a satisfactory result was obtained with a low concentration of inhibitors (Dai et al., 2021). In addition, many kinds of byproducts such as acetic acid and glycerol were generated inductively from *I. orientalis* HN-1 under the stress of inhibitors. These factors would lead to a reduction of ethanol yield. In order to achieve higher ethanol yield, the ethanol production strain should be strengthened in the future.

## Conclusion

A lignocellulosic ethanol production from residual tobacco stalk by integrating sulfuric acid presoak and ICSE pretreatment was investigated in this study. The optimum concentration of sulfuric acid for presoak of tobacco stalk was obtained. Neither single dilute acid soaking nor single ICSE could achieve a satisfactory nicotine removal rate, but the integrated pretreatment methods could remove almost all the nicotine of tobacco stalk. Effective removal of nicotine is essential to ensure that a high glucose concentration is achieved in the enzymatic hydrolysis process, and finally, a satisfactory ethanol concentration and yield were achieved by *I. orientalis* HN-1 in the fermenter. This environment-friendly technology provided a promising option for tobacco stalk resource utilization for ethanol production. Tobacco residual could be an important alternative feedstock for future industrial ethanol production.

## Data Availability

The original contributions presented in the study are included in the article/Supplementary Material, and further inquiries can be directed to the corresponding authors.

## References

[B1] Adney B. Baker J. (1996). Measurement of Cellulase Activities. Lab. Anal. procedure 6, 1–11.

[B2] Akpinar O. Erdogan K. Bakir U. Yilmaz L. (2010). Comparison of Acid and Enzymatic Hydrolysis of Tobacco Stalk Xylan for Preparation of Xylooligosaccharides. LWT - Food Sci. Technol. 43, 119–125. 10.1016/j.lwt.2009.06.025

[B3] Cai J. Li B. Chen C. Wang J. Zhao M. Zhang K. (2016). Hydrothermal Carbonization of Tobacco Stalk for Fuel Application. Bioresour. Technol. 220, 305–311. 10.1016/j.biortech.2016.08.098 27589825

[B4] Dai L. Huang T. Jiang K. Zhou X. Xu Y. (2021). A Novel Recyclable Furoic Acid-Assisted Pretreatment for Sugarcane Bagasse Biorefinery in Co-production of Xylooligosaccharides and Glucose. Biotechnol. Biofuels. 14, 35. 10.1186/s13068-021-01884-3 33531058 PMC7856728

[B5] Dash A. K. Wong S.-T. (1996). Liquid Chromatographic Method for the Determination of Nicotine in Pharmaceutical Formulations. J. Chromatogr. A 749, 81–85. 10.1016/0021-9673(96)00369-X

[B6] Erat M. Ciftci M. Gumustekin K. Gul M. (2007). Effects of Nicotine and Vitamin E on Glutathione Reductase Activity in Some Rat Tissues *In Vivo* and *In Vitro* . Eur. J. Pharmacol. 554, 92–97. 10.1016/j.ejphar.2006.10.008 17113070

[B7] Eveleigh D. E. Mandels M. Andreotti R. Roche C. (2009). Measurement of Saccharifying Cellulase. Biotechnol. Biofuels. 2, 21. 10.1186/1754-6834-2-21 19723298 PMC2753563

[B8] Falco A. M. Bevins R. A. (2015). Individual Differences in the Behavioral Effects of Nicotine: A Review of the Preclinical Animal Literature. Pharmacol. Biochem. Behav. 138, 80–90. 10.1016/j.pbb.2015.09.017 26410616 PMC4623936

[B9] Gan Z. Zhuang Q. (2002). Investigation of the Effect of Nicotine on the Activity of Lactate Dehydrogenase. Chin. J. Anal. Chem. 30, 385–387. 10.1016/S0956-5663(01)00270-6

[B10] Guo G.-N. Cai B. Li R. Pan X. Wei M. Zhang C. (2019). Enhancement of Saccharification and Ethanol Conversion from Tobacco Stalks by Chemical Pretreatment. Biomass Conv. Bioref. 11, 1085–1092. 10.1007/s13399-019-00478-2

[B11] Henry J. B. Vann M. C. Lewis R. S. (2019). Agronomic Practices Affecting Nicotine Concentration in Flue‐Cured Tobacco: A Review. Agron.j. 111, 3067–3075. 10.2134/agronj2019.04.0268

[B12] Herbert G. M. J. Krishnan A. U. (2016). Quantifying Environmental Performance of Biomass Energy. Renew. Sustain. Energ. Rev. 59, 292–308. 10.1016/j.rser.2015.12.254

[B13] Huang C. Sun R. Chang H. Yong Q. Jameel H. Phillips R. (2019). Production of Dissolving Grade Pulp from Tobacco Stalk through SO_2_-Ethanol-Water Fractionation, Alkaline Extraction, and Bleaching Processes. BioResources 14, 5544–5558.

[B14] Liang M. Yang T. Zhang G. Zhang K. Wang L. Li R. (2021). Effects of Hydrochloric Acid Washing on the Structure and Pyrolysis Characteristics of Tobacco Stalk. Biomass Conv. Bioref. 10.1007/s13399-021-01616-5

[B15] Morales M. Quintero J. Conejeros R. Aroca G. (2015). Life Cycle Assessment of Lignocellulosic Bioethanol: Environmental Impacts and Energy Balance. Renew. Sustain. Energ. Rev. 42, 1349–1361. 10.1016/j.rser.2014.10.097

[B16] Qin Z. Sun M. Luo X. Zhang H. Xie J. Chen H. (2018). Life-cycle Assessment of Tobacco Stalk Utilization. Bioresour. Technol. 265, 119–127. 10.1016/j.biortech.2018.05.110 29885497

[B17] Sluiter A. Hames B. Ruiz R. Scarlata C. Sluiter J. Templeton D. (2008). Determination of Structural Carbohydrates and Lignin in Biomass. Lab. Anal. procedure 1617, 1–16.

[B18] Sophanodorn K. Unpaprom Y. Whangchai K. Homdoung N. Dussadee N. Ramaraj R. (2020). Environmental Management and Valorization of Cultivated Tobacco Stalks by Combined Pretreatment for Potential Bioethanol Production. Biomass Conv. Bioref. 10.1007/s13399-020-00992-8

[B19] Su Y. Xian H. Shi S. Zhang C. Manik S. M. N. Mao J. (2016). Biodegradation of Lignin and Nicotine with white Rot Fungi for the Delignification and Detoxification of Tobacco Stalk. BMC Biotechnol. 16, 81. 10.1186/s12896-016-0311-8 27871279 PMC5117543

[B20] Sun D. Sun S.-C. Wang B. Sun S.-F. Shi Q. Zheng L. (2020). Effect of Various Pretreatments on Improving Cellulose Enzymatic Digestibility of Tobacco Stalk and the Structural Features of Co-produced Hemicelluloses. Bioresour. Technol. 297, 122471. 10.1016/j.biortech.2019.122471 31787511

[B21] Swanson J. A. Lee J. W. Hopp J. W. (1994). Caffeine and Nicotine: A Review of Their Joint Use and Possible Interactive Effects in Tobacco Withdrawal. Addict. Behaviors 19, 229–256. 10.1016/0306-4603(94)90027-2 7942243

[B22] Trigo J. M. Foll B. L. (2016). Inhibition of Monoacylglycerol Lipase (MAGL) Enhances Cue-Induced Reinstatement of Nicotine-Seeking Behavior in Mice. Psychopharmacology 233, 1815–1822. 10.1007/s00213-015-4117-5 26490035

[B23] Wang F. Dong H. Hassanpour M. Zhang K. Xie H. Zhang H. (2020). Glycerol-assisted One-step Instant Catapult Steam Explosion Enhances Enzymatic Digestibility of Corn stover. Ind. Crops Prod. 157, 112907. 10.1016/j.indcrop.2020.112907

[B24] Wang Q. Xu F.-Z. An L.-L. Xiang H.-Y. Zhang W.-H. Liu G.-S. (2019). Molecular Characterization of a New Recombinant brassica Yellows Virus Infecting Tobacco in China. Virus Genes 55, 253–256. 10.1007/s11262-019-01636-4 30697673

[B25] Xie H. Li Z. Wang Z. Mao G. Zhang H. Wang F. (2020). Instant Catapult Steam Explosion: A Rapid Technique for Detoxification of Aflatoxin-Contaminated Biomass for Sustainable Utilization as Animal Feed. J. Clean. Prod. 255, 120010. 10.1016/j.jclepro.2020.120010

[B26] Yalcin E. B. Tong M. Gallucci G. de la Monte S. M. (2018). Effects of Tobacco Nicotine-Derived Nitrosamine Ketone (NNK) Exposures on Brain Alcohol Metabolizing Enzyme Activities. Drug Metab. Lett. 12, 117–124. 10.2174/1872312812666180611115418 29886839 PMC9964543

[B27] Yu Z. Zhang B. Yu F. Xu G. Song A. (2012). A Real Explosion: The Requirement of Steam Explosion Pretreatment. Bioresour. Technol. 121, 335–341. 10.1016/j.biortech.2012.06.055 22858504

[B28] Yuan Z. Wei W. Wen Y. Wang R. (2019). Comparison of Alkaline and Acid-Catalyzed Steam Pretreatments for Ethanol Production from Tobacco Stalk. Ind. Crops Prod. 142, 111864. 10.1016/j.indcrop.2019.111864

[B29] Zhang H. Wang L. Dai Z. Zhang R. Chen C. Liu G. (2020). Effect of Organic Loading, Feed-To-Inoculum Ratio, and Pretreatment on the Anaerobic Digestion of Tobacco Stalks. Bioresour. Technol. 298, 122474. 10.1016/j.biortech.2019.122474 31865253

[B30] Zhao J. Zhang X. Lei W. Ji X. Zhou X. Xu Y. (2020). Mannonic Acid and Bio-Ethanol Production from Konjac Using a Two-step Bioprocess with *Candida Shehatae* and *Gluconobacter Oxydans* . J. Renew. Mater. 8, 79–88. 10.32604/jrm.2020.08761

[B31] Zhong W. Zhu C. Shu M. Sun K. Zhao L. Wang C. (2010). Degradation of Nicotine in Tobacco Waste Extract by Newly Isolated Pseudomonas Sp. ZUTSKD. Bioresour. Technol. 101, 6935–6941. 10.1016/j.biortech.2010.03.142 20434329

[B32] Zhou X. Hua X. Huang L. Xu Y. (2019). Bio-Utilization of Cheese Manufacturing Wastes (Cheese Whey Powder) for Bioethanol and Specific Product (Galactonic Acid) Production via a Two-step Bioprocess. Bioresour. Technol. 272, 70–76. 10.1016/j.biortech.2018.10.001 30312870

